# Effect of reducing agents on the synthesis of anisotropic gold nanoparticles

**DOI:** 10.1186/s40580-021-00296-1

**Published:** 2022-01-17

**Authors:** Sunghoon Yoo, Dong Hwan Nam, Thangjam Ibomcha Singh, Gyu Leem, Seunghyun Lee

**Affiliations:** 1 Department of Applied Chemistry, Hanyang University ERICA, Ansan, 15588 Republic of Korea; 2 Department of Chemical and Molecular Engineering, Hanyang University ERICA, Ansan, 15588 Republic of Korea; 3 Center for Bionano Intelligence Education and Research, Hanyang University ERICA, Ansan, 15588 Republic of Korea; 4 Department of Chemistry, College of Environmental Science and Forestry, State University of New York, 1 Forestry Drive, Syracuse, NY 13210 USA; 5 The Michael M. Szwarc Polymer Research Institute, 1 Forestry Drive, Syracuse, NY 13210 USA

**Keywords:** Gold nanorods, Aspect ratio, Ascorbic acid, Hydroquinone, Reducing agents, Seed-mediated

## Abstract

The seed-mediated method is a general procedure for the synthesis of gold nanorods (Au NRs), and reducing agents such as ascorbic acid (AA) and hydroquinone (HQ) are widely used for the growth process. Further, they are mild reducing agents; however, when AA is used, controlling the size of Au NRs with a higher aspect ratio (localized surface plasmon resonance (LSPR) peak, λ_Lmax_ > 900 nm) is challenging because it results in a faster growth rate of Au NRs. In contrast, when HQ is used, Au NRs with a higher aspect ratio can be synthesized as it slows down the growth rate of the Au NRs and greatly enhanced the λ_Lmax_. However, the increase in λ_Lmax_ is still needs not satisfactory due to the limited enhancement in the aspect ratio of Au NRs due to utilization of single reducing agent. The growth kinetics of the Au NRs can be modulated by controlling the reducing power of the reducing agents. In such scenario, judicious use of two reducing agents such as AA and HQ simultaneously can help us to design Au NRs of higher aspect ratio in a controlled manner due to the optimum growth rate resulting from the combined effect of both the reducing agents. In this study, we investigated the effect of the two reducing agents by controlling the volume ratios. When the growth solution contains both the reducing agents, the growth of Au NRs is first initiated by the fast reduction of Au^3+^ to Au^+^ due to stronger reducing power of the AA and when the AA in the growth solution is completely utilized, further growth of the Au NRs continues as a result of the HQ thereby resulting to high aspect ratio Au NRs. Consequently, the LSPR peak (λ_Lmax_ > 1275 nm) can be tuned by controlling the volume ratios of the reducing agents.

**Supplementary Information:**

The online version contains supplementary material available at 10.1186/s40580-021-00296-1.

## Introduction

Gold nanoparticles are extensively studied noble metal nanoparticles owing to their applications, including in biosensors and photocatalysis, cancer therapy, drug delivery, and antimicrobial activities. In addition, gold nanoparticles are applied in various analytical performance enhancements, including surface-enhanced Raman Scattering, owing to their excellent physical/chemical properties such as stability, biocompatibility, antioxidative, target-specific easy surface modifications, and localized surface plasmon resonance (LSPR) effect [1–20]. In particular, the LSPR effect induces photothermal and photoacoustic properties in colloidal Au nanoparticles upon irradiation with light of specific wavelengths, rendering them potential candidates for hyperthermic treatments and various medical imaging applications [21–25]. Therefore, tuning the shape and size of gold nanoparticles is crucial for modulating their LSPR, enhancing their photothermal and photoacoustic properties, and for utilizing light at various wavelengths, particularly in the near-infrared region, as it contributes the maximum in the solar spectrum. Among Au nanoparticles of various morphologies, Au NRs attract interest because of the existence of both longitudinal and transverse resonance resulting from their corresponding length and diameter. Further, the LSPR spectral location can be adjusted to the near-infrared radiation (NIR) spectrum by tuning the aspect ratio of the Au NRs. Accordingly, studies to control the aspect ratio of Au NRs have been actively progressing as increasing the aspect ratio will also increase the SPR absorption maximum (∆λ_max_) since they are directly proportional to each other, according to Eq. (1) [26]:

1 $$\Delta \lambda_{{{\text{max}}}} \, = \,{95}\, \times \,{\text{Aspect ratio}}\, + \,{42}0$$

The synthesis of Au NRs based on a seed-mediated method was first developed by Murphy et al. [27] and Nikoobakht and El-Sayed. [28] In this synthesis method, following the synthesis of Au seed particles with a size of 2–3 nm, they are grown into Au NRs in a growth solution. Sodium borohydride was used as a strong reducing agent without any additional reagents for seed particle formation. Subsequently, the seed particles can be grown into Au NRs using a relatively mild reducing agent owing to the presence of seed particles in the growth solution. The control of the aspect ratio of Au NRs depends on the reducing agents used in the growth solution. The rate at which Au ions are reduced during the growth step depends on the reducing power of the reducing agent. Ascorbic acid (AA) and hydroquinone (HQ) are representative reducing agents used in the synthesis of Au NRs. The Au NR synthesis method introduced by El-Sayed and Murphy used AA as a reducing agent, and since then, many studies have been conducted using AA [27, 28]. Au NRs using AA as a reducing agent have a LSPR peak (λ_Lmax_) below 850 nm and an aspect ratio of 3.5–4. Further, Zubarev et al. [29] introduced a method using HQ as a reducing agent, and this method controlled the λ_Lmax_ from 850 to 1250 nm and adjusted the aspect ratio of 6–8. In both synthesis methods, the control of λ_Lmax_ is dependent on the reducing power of the reducing agents used. AA and HQ are mild reducing agents with a standard reduction potential of − 0.081 [30] and 0.714 V, [31] respectively. AA, which has a relatively stronger reducing power, reduces Au^3+^ to Au^+^ faster than HQ, so the growth rate of Au NRs is faster and results in the non-uniform growth of Au NRs. In contrast, the rate of reduction of Au^3+^ to Au^+^ proceeds slowly with HQ; consequently, the aspect ratio is easily adjusted by controlling the rate of reduction. However, the combined effect of both the reducing agents on the synthesis of Au NRs regarding the aspect ratio and uniformity has not been explored till date.

Initially, AA may cause the reduction of some Au ions to grow into relatively short Au NRs because of its stronger reducing power than HQ; subsequently, HQ can gradually reduce the remaining Au ions after AA is completely utilized. This can be achieved by controlling the amounts of AA and HQ. In this study, we investigated the effect of using both AA and HQ as reducing agents on the aspect ratio of Au NRs, and the effect of the combination of the reducing agents mixed at different ratios, on the growth of the Au NRs. This study shows that Au NRs with a high aspect ratio can be obtained with uniformity by controlling the volumes of AA and HQ, and the λ_Lmax_ can be shifted to the NIR region to a maximum of approximately 1300 nm, suggesting its potential application in NIR-based photothermal applications.

## Experimental details

### Materials

Silver nitrate (AgNO_3,_ 99.85%), hexadecyltrimethylammonium bromide (CTAB, 99 + %), and HQ (99.5%) were purchased from Acros Organics. L-AA (AA, $\ge$ 99.0%), sodium borohydride ($\ge$ 98.0%), and gold (III) chloride trihydrate (HAuCl_4_, ≥ 99.9%), were purchased from Sigma-Aldrich. All solutions were prepared fresh for each characterization, and the gold (III) chloride solution was used as a stock solution.

### Characterization

The UV–Vis-NIR absorption spectra were obtained using a JASCO V-770 spectrophotometer. Since the synthesized Au NRs were highly concentrated, they were diluted with deionized water in a ratio of 1:2 to record UV–Vis-NIR spectra. Transmission electron microscopy (TEM) images were obtained using a JEOL JEM-3010 (Core-facility for Bionano Materials in Gachon University). For TEM measurements, CTAB was removed by centrifugation twice.

### Synthesis of gold seed particles

The solution for the synthesis of Au seed nanoparticles was prepared as previously reported, with slight modifications [18, 27–29]. Typically, 0.5 mL of 0.01 M HAuCl_4_ was added to 9.5 mL of 0.1 M CTAB. Then, a 0.01 M ice-cold NaBH_4_ aqueous solution was slowly injected into the seed solution. The color of the seed solution changed from yellow to light brown. The seed solution was incubated for at least 30 min in a water bath maintained at 40 °C and used within 2–5 h.

### Synthesis of Au NRs

The growth solution for the synthesis of Au NRs was prepared with a slight modification using previously reported methods [27–29]. Initially, 9.5 mL of 0.1 M CTAB, 0.5 mL of 0.01 M HAuCl_4_, and 0.05 mL of 0.1 M AgNO_3_ were added; when the reducing agent was further added into the solution, the solution became colorless owing to the reduction of Au ions. If the growth solution did not become colorless, it signified that the Au ions were not completely reduced. The minimum volumes of 100 mM HQ and 100 mM AA required to reduce Au^3+^ to Au^+^ were 300 and 70 μL, respectively. If both the reducing agents were mixed at the same concentration, the Au NRs would grow only by AA since AA has a relatively stronger reducing power than HQ, and confirming the effect of the two reducing agents could be challenging. To study the reducing effect of both AA and HQ, different amounts of AA and HQ were used, as listed in Additional file 1: Table S1. Finally, 160 µL of the Au seed solution was added to the growth solution and then kept in a water bath maintained at 40 °C. The growth time varied depending on the mixed volume of the reducing agents, and when the growth of Au NRs was completed, the solution became dark brown. In the similar process, Au NRs were also prepared using only HQ for comparison.

We also synthesized Au NRs using only ascorbic acid following previously reported method from Murphy’s group with slight modifications [32]. Typically, 4.75 mL of 0.1 M CTAB, 0.2 mL of 0.01 M HAuCl_4_, and 0.03 mL of 0.01 M AgNO_3_ were added in order followed by addition of 0.03 mL of 0.1 M AA to prepare the growth solution. Finally, 0.06 mL of Au seed solution was added into the growth solution and kept in a water bath maintained at 40 °C for 1 h.

## Results and discussion

**Fig. 1:**
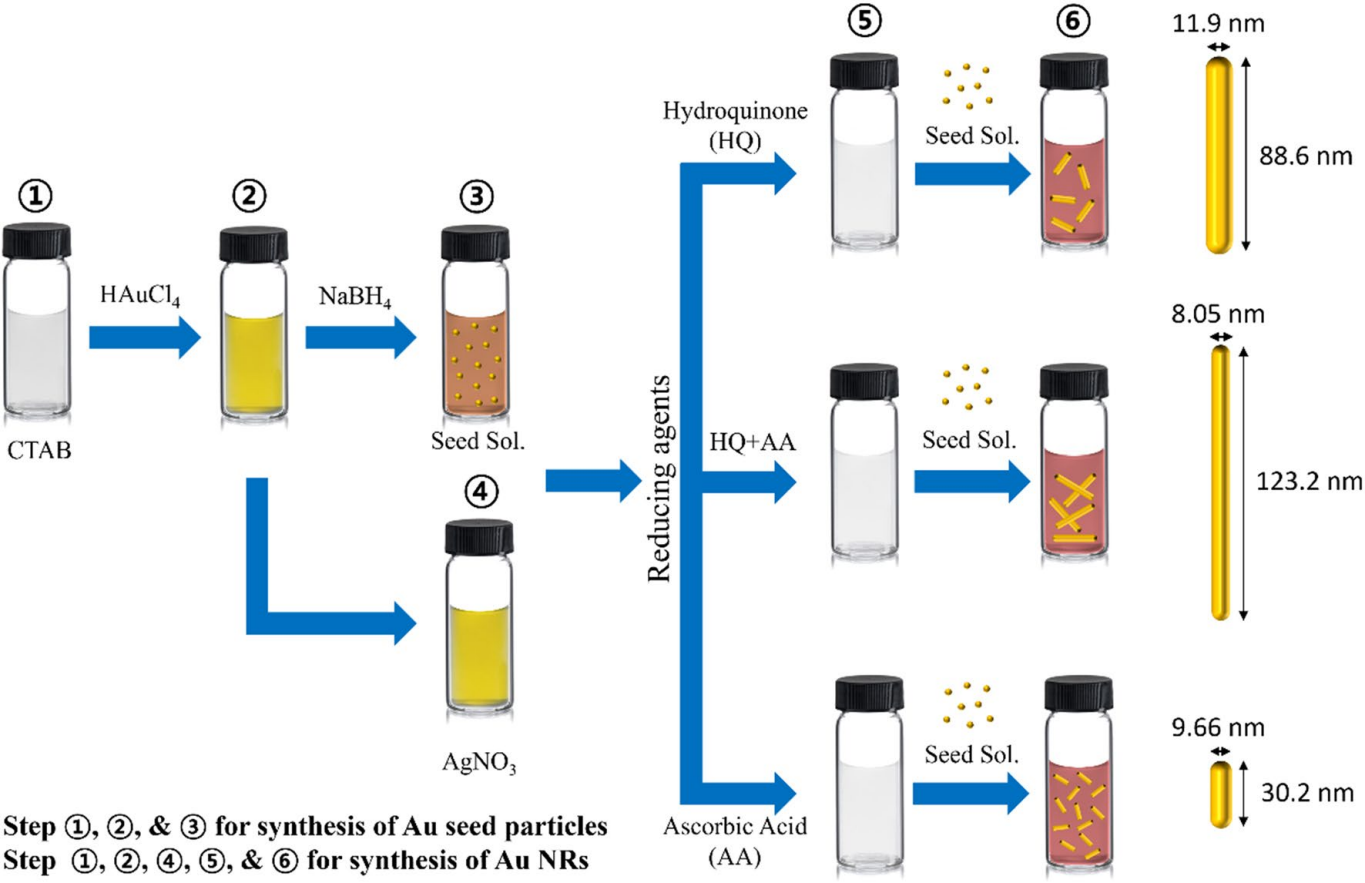
Schematic of the synthesis of Au NRs via the seed-mediated method

Rod-shaped anisotropic Au NRs were synthesized using a seed-mediated growth method, as shown in Fig. 1. In the seed particle synthesis step, NaBH_4_, a strong reducing agent, was used to synthesize spherical particles with a size of 2–3 nm, by reducing Au ions. In contrast, in the Au NRs growth step, a mild reducing agent was used to grow Au NRs on the pre-synthesized seed particles. Therefore, selecting an appropriate reducing agent is essential so that Au NRs with the desired aspect ratio are synthesized during the growth step.

AA and HQ are used as reducing agents during the growth stage. When Au NRs were grown using AA, the rate at which Au^3+^ was reduced to Au^+^ was faster; consequently, the growth rate of Au NRs was faster than when HQ was used as the reducing agent. Therefore, relatively short Au NRs were formed when AA was used. In contrast, Au ion reduction occurred slowly when HQ was used; therefore, the growth rate of the Au NRs was slow, resulting in the growth of longer Au NRs.

**Fig. 2:**
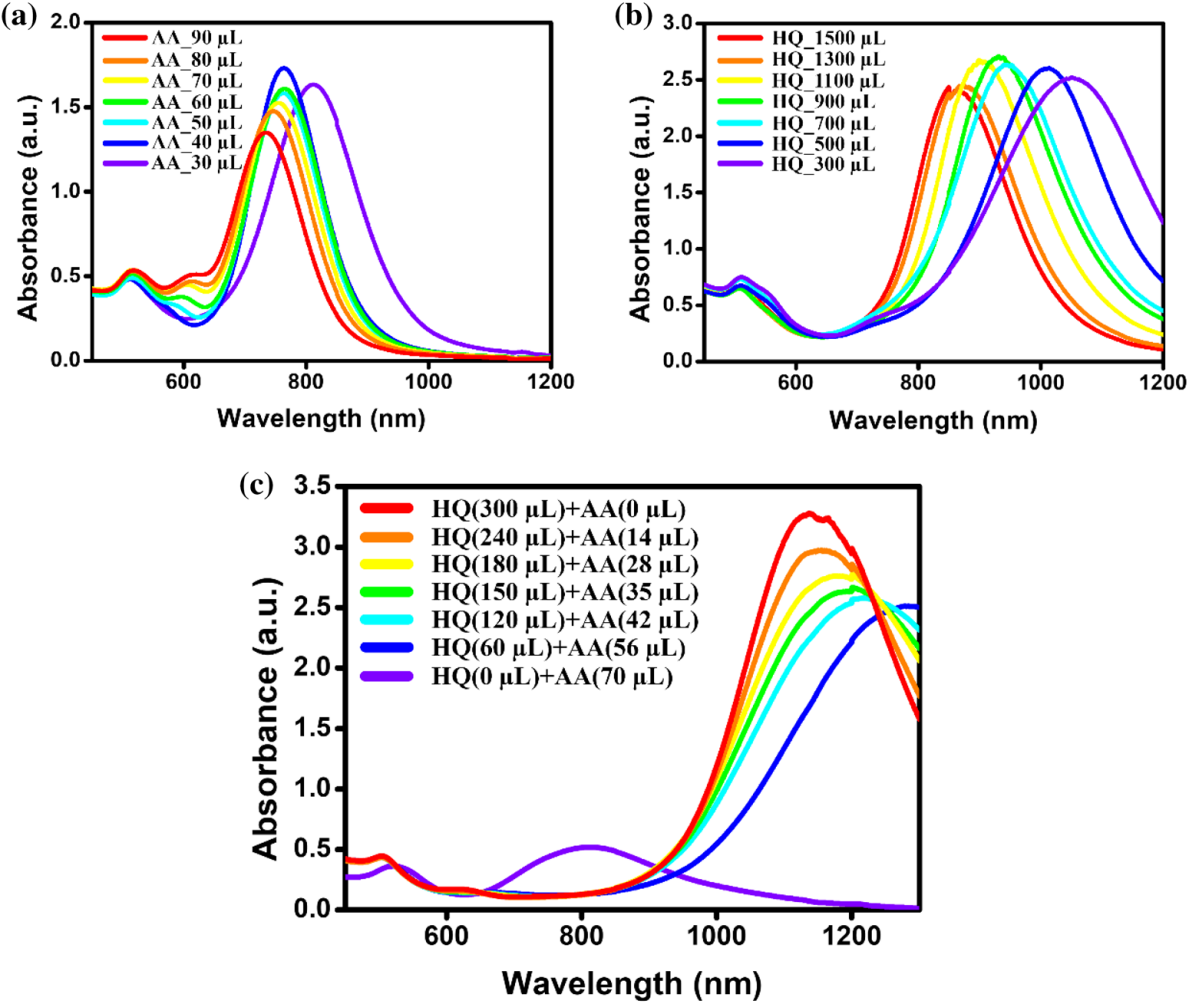
Absorption spectra of Au NRs synthesized by controlling the volume of **a** AA and **b** HQ as reducing agents. **c** Absorbance spectra of Au NRs prepared under the combined effect of HQ and AA, mixed at different volume ratios

The UV–Vis-NIR spectra of the Au NRs prepared at different volumes of the reducing agents, AA and HQ, are shown in Fig. 2a, b, respectively. In Fig. 2a, λ_Lmax_ of the Au NRs shifted from 734 nm to a maximum of 812 nm i,e. λ_Lmax_ = 78 nm only on changing the volume of AA from 90 to 30 μL. While, in the case of using HQ as shown in Fig. 2b, λ_Lmax_ shifted significantly from 850 to 1066 nm (λ_Lmax_ = 216) on changing the volume of the HQ. This significant change in λ_Lmax_ in the case of using HQ compared to those of AA may be due to the difference in the aspect ratio of the prepared Au NRs in each cases. To confirm this, we investigated the Au NRs using the TEM technique as shown in Additional file 1: Figs. S1 and S2., Analysis of the TEM images revealed that the changes in length and diameter were also not much in the case of Au NRs prepared with AA only and resulted to low aspect ratio (3.14) (Additional file 1: Fig. S1), while in the case of HQ in Additional file 1: Fig. S2, the change in length and diameter were changed significantly and resulted to high aspect ratio (7.43). This increase in the aspect ratio of Au NRs in the case of using HQ is responsible for increasing λ_Lmax_ compared to those of Au NRs using the AA (Fig. 2b–c). This is attributed to the difference in the growth rate of the Au NRs originating from the difference in the reducing power of the reducing agents. Since HQ is a weaker reducing agent than AA, the lower the volume of HQ, the slower the reduction of Au^3+^ to Au^+^. Therefore, the growth of Au NRs will occur slowly, resulting in the synthesis of high-aspect-ratio Au NRs that can absorb light of longer wavelengths. As the volume of HQ increases, the λ_Lmax_ shifts to a shorter wavelength because the Au^3+^ is rapidly reduced to Au^+^, which does not provide sufficient time for the Au NRs to grow. This indicates that the growth of Au NRs can be controlled by reducing the power of the reducing agent. Further, we investigated the effect of controlling the reducing power of the reducing agent for the growth of the Au NRs by mixing different volume ratios of the two reducing agents. When the AA was used, it was not easy to adjust the λ_Lmax_, and when HQ was used, the λ_Lmax_ could not be adjusted after 1066 nm. However, when both the reducing agents were used, the λ_Lmax_ could be adjusted up to 1250 nm. UV–Vis-NIR absorption spectra of the Au NRs prepared by mixing different volume ratios of AA and HQ are shown in Fig. 2c.

**Fig. 3:**
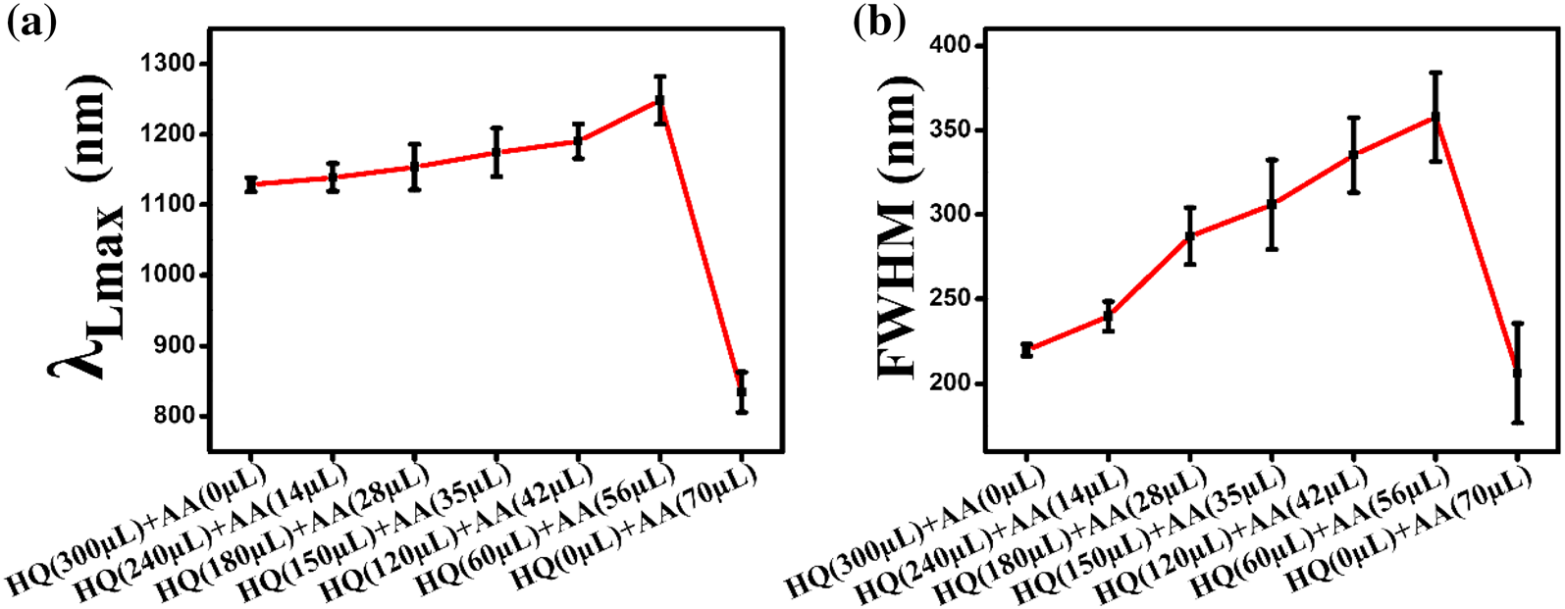
Change in **a** λ_Lmax_ and **b** The full-width-half-maximum (FWHM) of UV–Vis-NIR absorption spectrum of Au NRs for each mixed volume of HQ and AA

In Fig. 2c, the λ_Lmax_ is red shifted as the proportion of AA in the reducing solution mixture increases, which can also be confirmed in Fig. 3a. The full-width-half-maximum (FWHM) of the UV–Vis-NIR absorption spectrum, which represents the degree of uniformity of the particles, increased as the proportion of AA increased (Fig. 3b). This is owing to the faster rate of the reaction and the lesser uniformity resulting from the higher proportion of AA.

**Fig. 4:**
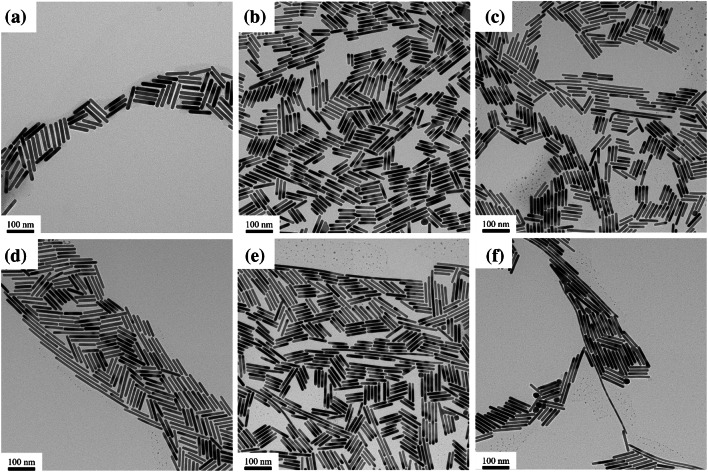
TEM images of the Au NRs prepared by mixing different volumes of HQ and AA: **a** HQ(300 μL) + AA(0 μL), **b** HQ(240 μL) + AA(14 μL), **c** HQ(180 μL) + AA(28 μL), **d** HQ(150 μL) + AA(35 μL), **e** HQ(120 μL) + AA(42 μL), and **f** HQ(60 μL) + AA(56 μL)

The TEM images of Au NRs prepared by mixing different volumes of HQ and AA are shown in Fig. 4. When the mixed volumes of HQ and AA were 0 and 70 μL, respectively, the yield of the Au NRs was not considered since it was significantly less. As the volume of AA increased from 0 to 56, the uniformity of Au NRs decreased. Au NRs with a high aspect ratio of several hundred nanometers were observed in the TEM images, as shown in Fig. 4a–f. This observation confirms that the aspect ratio of the Au NRs can be enhanced by controlling the reducing power of the reducing agent during the growth process. Further, this result is in agreement with the UV–Vis NIR absorption spectra in Figs. 2 and 3. However, this result is different from the anticipation that an increase in AA would quickly reduce Au^3+^ to Au^+^, which would not provide enough time for the Au NRs to grow.

**Fig. 5:**
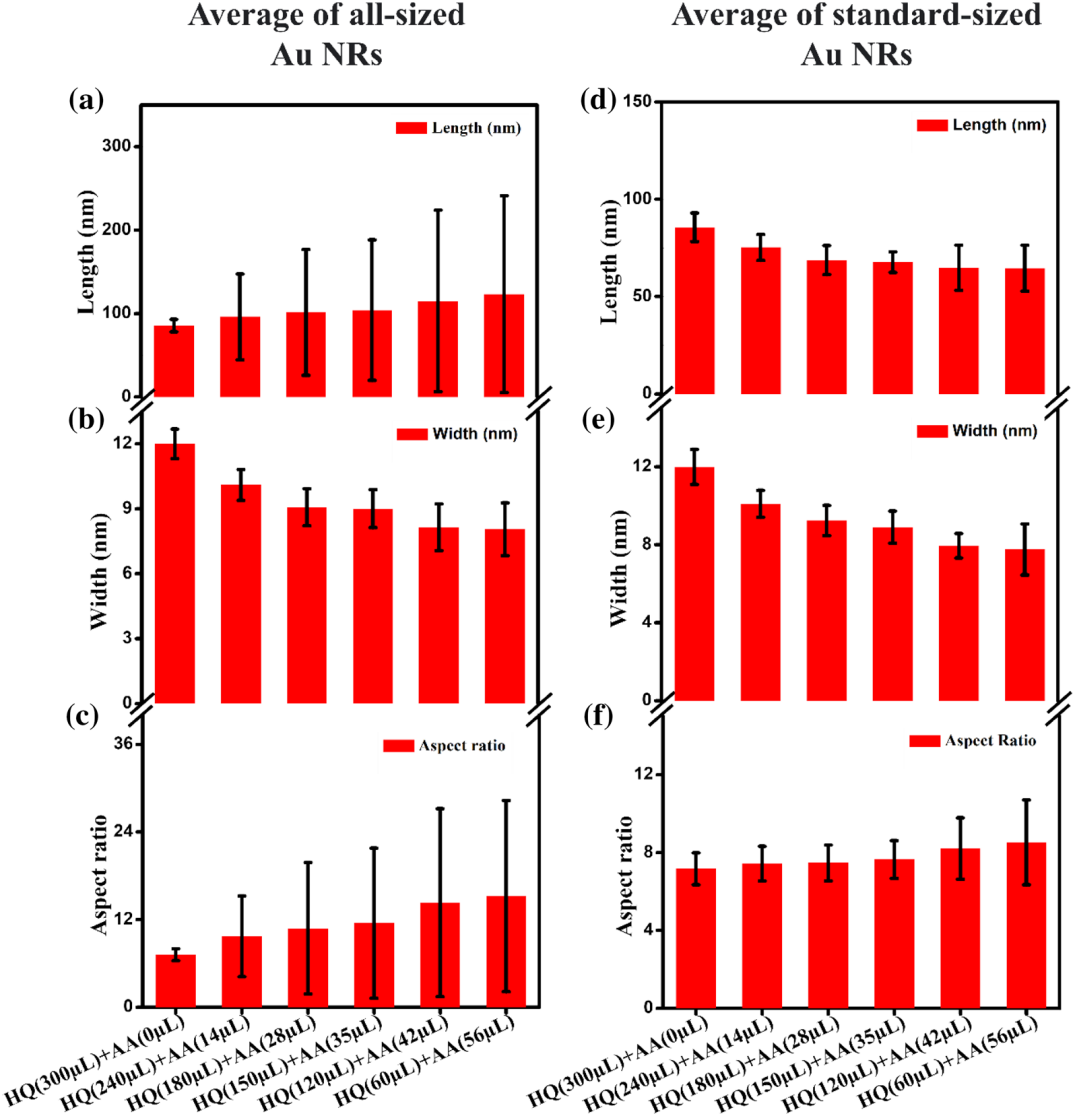
Average of **a** length, **b** diameter, and **c** aspect ratio of all-sized Au NRs. Average of **d** length, **e** diameter, and **f** aspect ratio of standard-sized Au NRs

The average length, width, and aspect ratio of Au NRs of all sizes by mixing different volumes of HQ and AA—HQ(300 µL) + AA(0 µL), HQ(240 µL) + AA(14 µL), HQ(150 µL) + AA(28 µL), HQ(120 µL) + AA(35 µL), HQ(60 µL) + AA(56 µL), are shown in Fig. 5a–c. As the volume of AA increased, the average length and aspect ratio of the Au NRs increased (Fig. 5a–c); however, the error bars also gradually increased. The increase in the error bars suggests that as the volume of AA increases, the growth rate of the Au NRs increases, and the uniformity decreases. The average length, width, and aspect ratio of the standard-sized Au NRs, excluding those Au NRs of exceptional length prepared by mixing different volumes of AA and HQ, are shown in Fig. 5d–f. As the volume of AA decreased and that of HQ increased, the average length and diameter of all the Au NRs decreased (Fig. 5d–e). When both reducing agents are present, AA, which has a relatively stronger reducing power, reduces Au ions faster than HQ. Therefore, Au reduced by AA was preferentially grown on the seed particles. The AA was consumed earliest; consequently, the volume of AA in the growth solution was not sufficient to reduce all Au ions. Therefore, only a few seed particles were initially grown into NRs. The remaining Au ions that were not reduced by AA were slowly reduced by HQ; subsequently, the remaining seed particles grew into NRs. Therefore, if both reducing agents are present in the Au NR growth solution, AA, which has a relatively stronger reducing power, preferentially reduces some Au ions, and eventually, HQ slowly reduces the remaining Au ions; accordingly, relatively long Au NRs are grown.

As a result, the rate of reduction of Au ions increased as the volume of AA increased. This resulted in a reduction in the average length and diameter of the standard-sized Au NRs (Fig. 5d–f) and the number of remaining seed particles decreased relatively. As the number of remaining seed particles decreases, the length of Au NRs becomes long because the reduced Au is relatively more attached to each seed particle, and the overall average length increases.

**Fig. 6:**
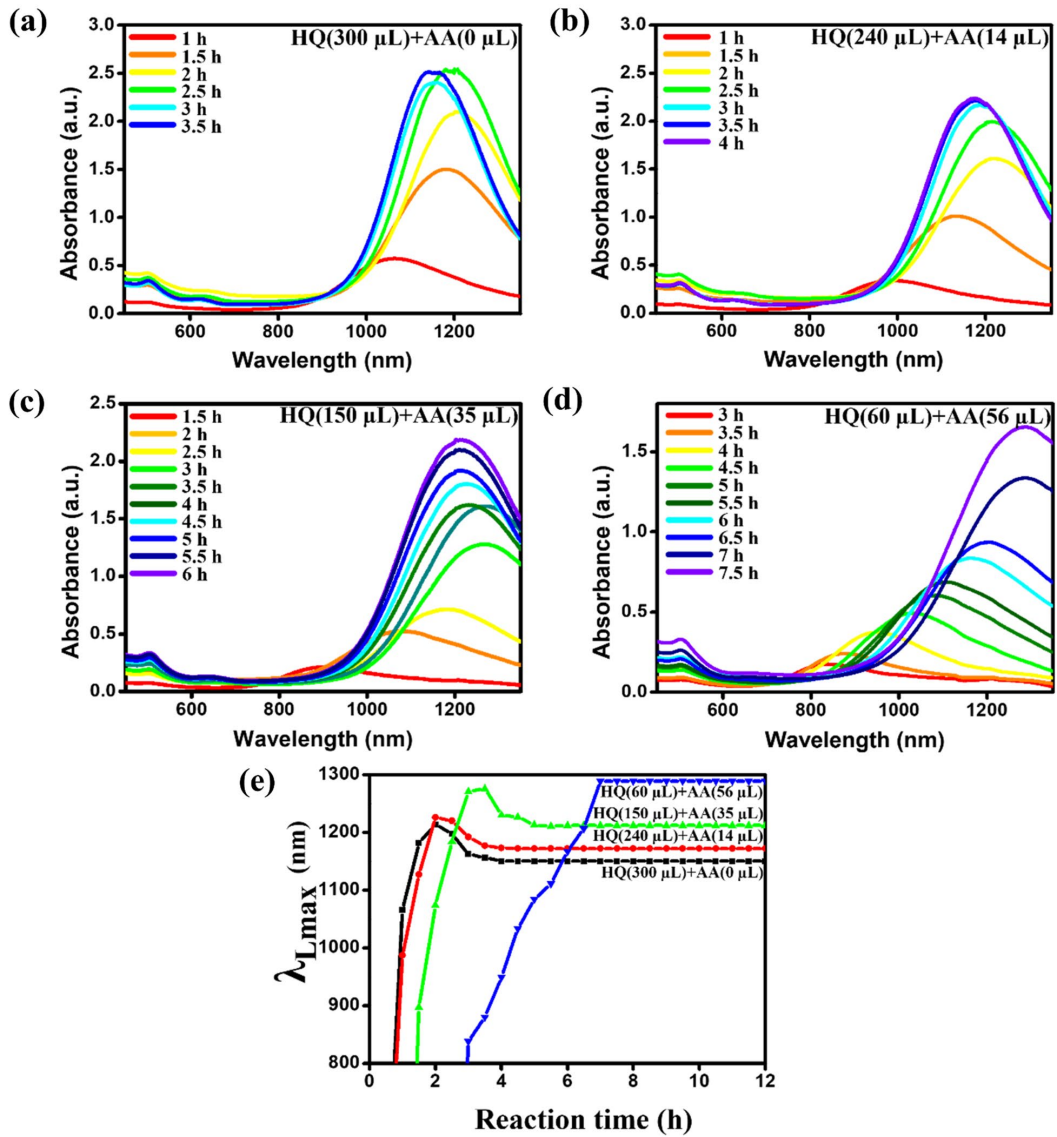
Evolution of the UV–Vis-NIR absorbance spectrum of Au NRs during their growth when the volume of HQ and AA ratio was **a** HQ(300 µL) + AA(0 µL), **b** HQ(240 µL) + AA(14 µL), **c** HQ(150 µL) + AA(28 µL), and **d** HQ(60 µL) + AA(56 µL) and the **e** corresponding graph of λ_Lmax_ w.r.t reaction time

Figure 6 shows the change of UV–Vis-NIR spectra of Au NRs at different mixed volumes of the reducing agents—HQ (300 µL) + AA (0 µL), HQ (240 µL) + AA (14 µL), HQ (150 µL) + AA (28 µL), and HQ (60 µL) + AA (56 µL) during each growth time. When the volumes of HQ and AA are 300 and 0; (Fig. 6a); 240 and 140 (Fig. 6b); 150 and 28 (Fig. 6c); 60 and 56 µL (Fig. 6d), the reaction times are 3, 3.5, 5.5, and 7.5 h, respectively. As shown in Fig. 5a, c, the average length and aspect ratio of the Au NRs increase as the volume of HQ decreases. Since the Au NRs require additional duration to become longer, the reaction time is the longest when the volumes of HQ and AA are 60 and 56 µL, respectively (Fig. 6d).

The standard reduction potentials of HQ and AA are 0.714 [31] and − 0.081 V [30], respectively, and HQ is a weaker reducing agent than AA. The pKa of AA is 4.12 [33] and that of HQ is 9.96, [34]. Since the pKa of AA is low, it is a relatively stronger acid, so it donates H^+^ and electrons better than HQ, which indicates that AA has a stronger reducing power than HQ. The growth mechanism of the Au NRs is that the Au particles reduced by the reducing agent in the growth solution are anisotropically attached to the seed particles and grow into Au NRs. HQ, which has relatively weak reducing power, reduces the Au ions slowly, and the reduced Au particles grow slowly on the seed particles, providing sufficient growth time to increase the length and diameter of the Au NRs. Since the growth of Au NRs is slow when HQ is used, the length and diameter of the Au NRs can be controlled by regulating the reaction rate by changing the volume of the reducing agent. However, when AA was used, the length and diameter of the Au NRs were shorter than those in the case of HQ, and controlling the aspect ratio was difficult because it did not provide sufficient growth time to grow into Au NRs. This study demonstrates the combined effect of AA and HQ reducing agents with different reducing powers, and their effect on the aspect ratio of the synthesized Au NRs and their UV–Vis NIR absorption spectra.

## Conclusions

In this study, we investigated the effect of combining reducing agents with different reducing powers during the growth step of Au NRs on the aspect ratio and UV–Vis NIR absorption spectra. Both AA and HQ are mild reducing agents; however, AA has a relatively stronger reducing power than HQ. Therefore, when HQ was used, the aspect ratio of Au NRs could be easily controlled, as it provides sufficient time to grow the Au NRs because of the slower reaction rate that can be controlled by adjusting the volume of the reducing agent. In contrast, controlling the aspect ratio was challenging when AA was used, even if the volume of the reducing agent was adjusted owing to fast reduction. Further, Au NRs synthesized by mixing the two reducing agents at different volume ratios showed high aspect ratios and a more redshifted λ_Lmax_ compared to those prepared using only AA or HQ. This is because Au ions were preferentially grown into Au NRs by AA initially, and then the remaining Au ions were grown into NRs by HQ in the later growth step.

## Supplementary Information

**Additional file 1: Table. S1** Amount of ascorbic acid and hydroquinone used when mixing reducing agent. **Fig. S1** **(a–c)** TEM images and average of **(d)** length, **(e)** diameter, and **(f) **aspect ratio when the volume of ascorbic acid is 30, 70, 90 uL. **Fig. S2** **(a–c)** TEM images and average of **(d)** length, **(e)** diameter, and **(f)** aspect ratio when the volume of hydroquinone is 300, 700, 1500 uL.

## Data Availability

Not applicable.

## Abbreviations

LSPR: Localized surface plasmon resonance
Au NRs: Gold nanorods
AA: Ascorbic acid
HQ: Hydroquinone
λ_Lmax_: LSPR peak
NIR: Near-infrared radiation
