# Supplementary material for: Effect of reducing agents on the synthesis of anisotropic gold nanoparticles
Source: Nano Converg. 2022 Jan 17;9:5. doi: 10.1186/s40580-021-00296-1 (PMC8762532; doi:10.1186/s40580-021-00296-1)
Supplement: Supplementary file 1 — Additional file 1: Table. S1 Amount of ascorbic acid and hydroquinone used when mixing reducing agent. Fig. S1 (a–c) TEM images and average of (d) length, (e) diameter, and (f) aspect ratio when the volume of ascorbic acid is 30, 70, 90 uL. Fig. S2 (a–c) TEM images and average of (d) length, (e) diameter, and (f) aspect ratio when the volume of hydroquinone is 300, 700, 1500 uL. [file 40580_2021_296_MOESM1_ESM.docx]

**Effect of reducing agents on the synthesis of anisotropic gold nanoparticles**

Sunghoon Yoo^1, 3, †^, Dong Hwan Nam^1, 3, †^, Thangjam Ibomcha Singh^2, 3^, Gyu Leem^4, 5*^, Seunghyun Lee^1, 2 ,3, 5*^

^1^ Department of Applied Chemistry, Hanyang University ERICA, Ansan, 15588, Republic of Korea

^2^ Department of Chemical and Molecular Engineering, Hanyang University ERICA, Ansan, 15588, Republic of Korea

^3^ Center for Bionano Intelligence Education and Research, Hanyang University ERICA, Ansan, 15588, Republic of Korea

^4^ Department of Chemistry, State University of New York, College of Environmental Science and Forestry, 1 Forestry Drive, Syracuse, NY, 13210, USA

^5^ The Michael M. Szwarc Polymer Research Institute, 1 Forestry Drive, Syracuse, NY, 13210, USA

*Corresponding authors: Seunghyun Lee and Gyu Leem

^†^ These authors contributed equally to this work.

Tel.: +82-31-400-5496

E-mail address: [leeshyun@hanyang.ac.kr](mailto:leeshyun@hanyang.ac.kr)


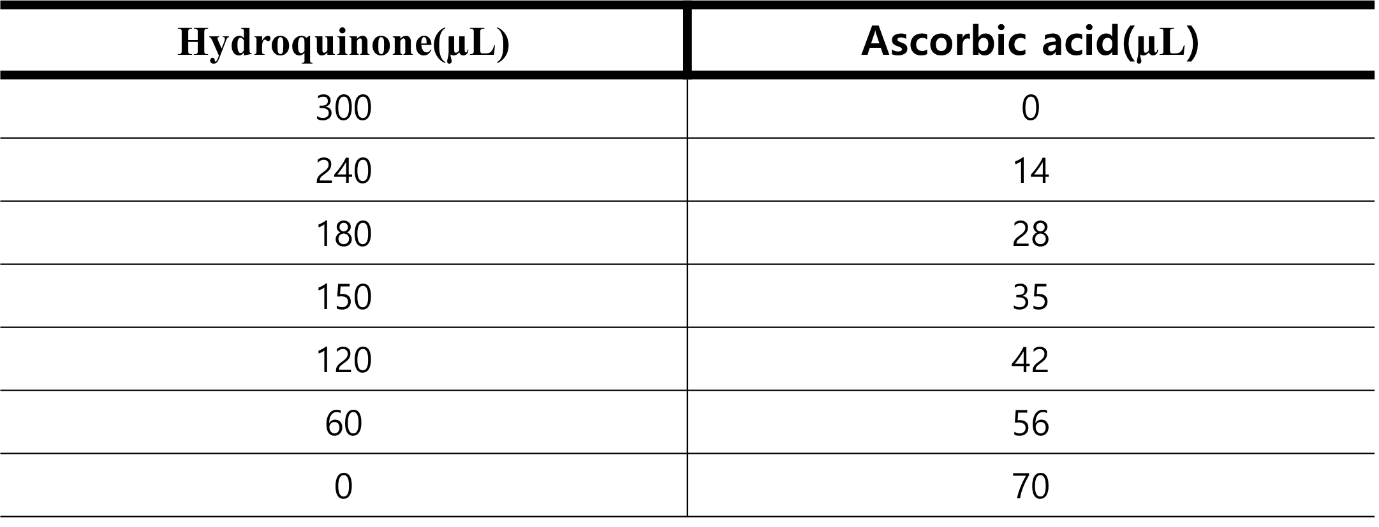


**Table. S1** Amount of ascorbic acid and hydroquinone used when mixing reducing agent.


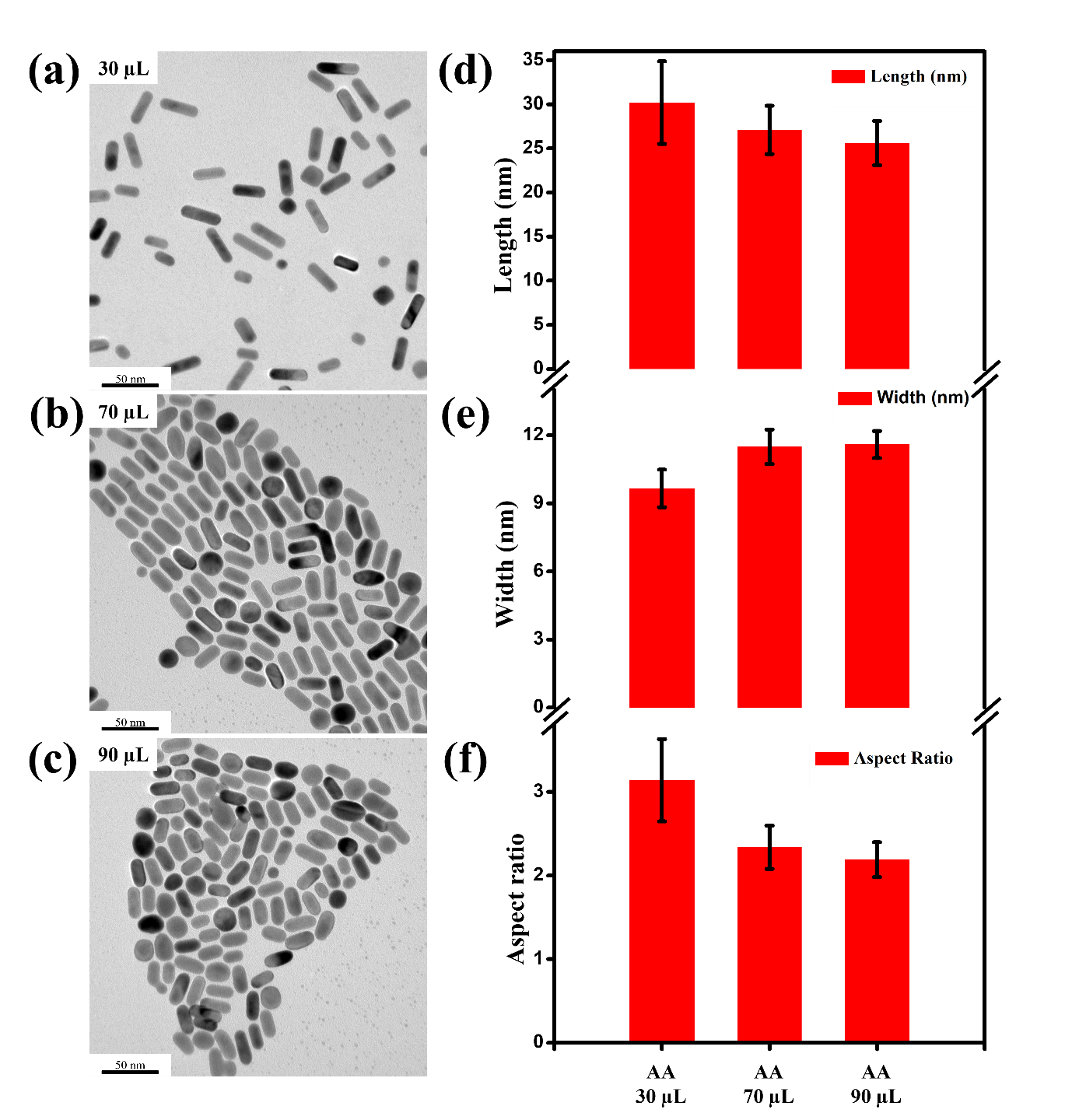


**Fig. S1** **(a)-(c)** TEM images and average of **(d)** length, **(e)** diameter, and **(f)** aspect ratio when the volume of ascorbic acid is 30, 70, 90 uL
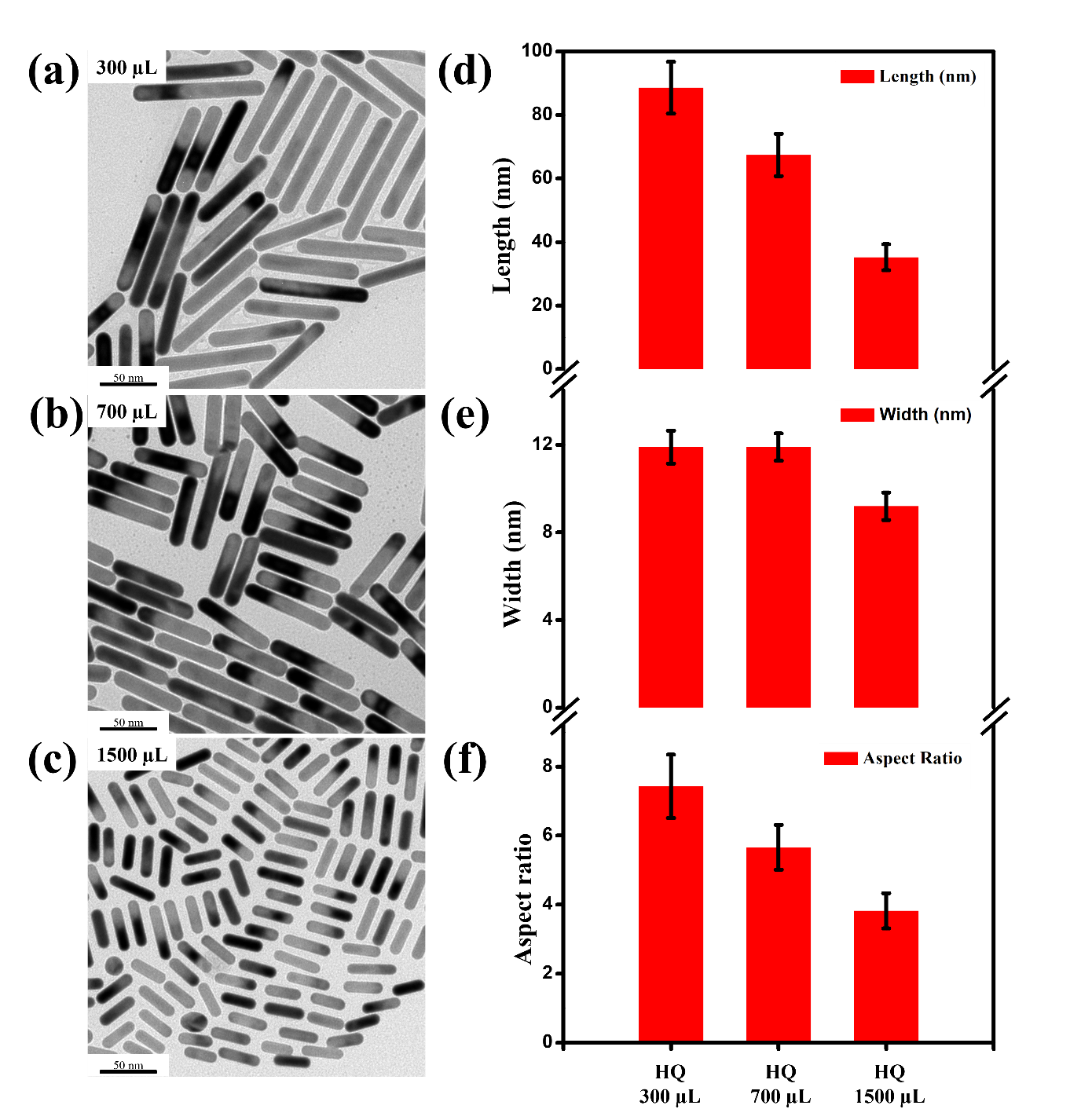
**Fig. S2** **(a)-(c)** TEM images and average of **(d)** length, **(e)** diameter, and **(f)** aspect ratio when the volume of hydroquinone is 300, 700, 1500 uL
